# Influence of lupin and faba bean on growth performance and nutrient digestibility in starter diets for broilers

**DOI:** 10.1016/j.psj.2026.107721

**Published:** 2026-08-29

**Authors:** Obright Hamungalu, M. Reza Abdollahi, Patrick C.H. Morel, Sonia Liu, Timothy J. Wester

**Affiliations:** a School of Agriculture and Environment, Massey University, Palmerston North, 4442, New Zealand; b Poultry Research Foundation, The University of Sydney, Camden, NSW, 2570, Australia

**Keywords:** Broilers, Lupin, Faba bean, Performance, nutrient digestibility

## Abstract

The effects of lupin and faba bean on growth performance, nutrient digestibility and gastrointestinal tract development of broilers fed starter diets were examined in a 21-day trial. Five diets based on soybean meal **(SBM)** or 100 or 200 g/kg Australian-grown lupin or faba bean were formulated and pelleted at 75°C. A total of 320, one-day-old male broilers (Ross 308) were allocated to 8 replicates per treatment, 8 birds per replicate. On d-21, birds from each replicate were euthanised, and ileal digesta was collected for nutrient digestibility. Pellet durability index, and hardness were lowest in diets containing 200 g/kg lupin or faba bean. Over the whole trial period, birds fed lupin diets had greater (*P<* 0.05) weight gain than those fed faba bean or SBM diet. Feed intake was lowest (*P<* 0.05) in faba bean, and feed conversion ratio (*P<* 0.05) poorest in birds fed SBM. Coefficient of apparent ileal digestibility **(CAID)** of nitrogen was greatest (*P<* 0.05) in grain legume diets, except that 100 g/kg faba bean was not different from SBM. The CAID of starch was greatest (*P<* 0.05) in lupin diets. Relative weight of gizzard and proventriculus of birds fed 200 g/kg faba bean, and 100 or 200 g/kg lupin diets were greater (*P<* 0.05) than those fed SBM diet. Gizzard and proventriculus weight of birds fed 100 g/kg faba bean were not different from birds fed SBM. These findings show that inclusion of lupin or faba bean up to 200 g/kg in broil er starter diets improved nutrient utilization and growth performance.

## Introduction

Broiler production is an important sector of agriculture, providing economical and high-quality animal protein to the growing human population throughout the globe (Biesek et al., 2020; OECD-FAO, 2021). Feed alone accounts for about 60-70% of the total cost of broiler production, with protein as a major expense (Noblet et al., 2022). Thus, exploring a cost-effective and sustainable source of protein is indispensable for sustaining economic production (Sadr et al., 2026). Many countries depend on imported soybean meal as a principal protein ingredient for broiler feed (Macelline et al., 2025; Sadr et al., 2026) largely because local soybean production cannot meet industry demands. Reliance on imported SBM presents challenges that include supply instability and carbon footprint associated with transportation (Nalle et al., 2012; Sadr et al., 2026). This necessitates exploration of locally available protein sources. Grain legumes, such as lupin and faba bean, are among the potential alternatives to SBM (Kaczmarek et al., 2016; Nalle et al., 2010a, b).

The amino acid **(AA)** profile of lupin and faba bean is low in sulphur containing AA compared to lysine (Diaz et al., 2006; Ravindran et al., 2002; Woyengo and Nyachoti, 2012). Nalle et al. (2011) reported lysine and methionine values of 14.9 – 15.9 and 2.44 – 2.55 g/kg in narrow-leafed lupin cultivars, respectively. In Adekoya and Adeola (2023), lysine and methionine contents were 15.8 and 1.7 g/kg in faba bean, respectively. Woyengo and Nyachoti (2012) found no difference in AA digestibility between faba bean and SBM. Also, Hamungalu et al. (2026b) reported that digestibility of AA in lupin was comparable to that of SBM. They noted greater digestibility of arginine, cysteine, glutamic acid and glycine in lupin than in SBM. Grain legumes also provide energy in broiler diets (Hamungalu et al., 2026a; Nalle et al., 2011).

Previous broiler performance studies involving lupin (Breytenbach and Ciacciariello, 2006; Kaczmarek et al., 2016; Nalle et al., 2011; Viveros et al., 2007) and faba bean (Ivarsson and Wall, 2017; Kopmels et al., 2020) reported contradictory results, particularly during the starter phase. Nalle et al. (2012) reported that inclusion of lupin at 200 g/kg in a starter diet supported feed intake **(FI)**, weight gain **(WG)** and feed conversion ratio **(FCR)** for 21-d old broilers. Similar observations were made in other studies (Eason et al., 1990; Nalle et al., 2011). However, Steenfeldt et al. (2003) reported an 8.4% reduction in WG and 0.16% increase in FCR for 21-d broilers fed a diet containing 200 g/kg lupin. Contradictory findings were reported in several other studies (Gous, 2011; Ivarsson and Wall, 2017; Koivunen et al., 2015; Nalle et al., 2010b) for broilers fed faba bean-based diets. Differences in outcome have been attributed largely to cultivar effect, diet formulation strategies, and level of anti-nutritional factors **(ANFs**) (Diaz et al., 2006; Nalle et al., 2010a; b), and climate and geographical location (Hughes et al., 1998; Saini et al., 1992). Therefore, because of the inherent variability in nutritional value of grain legumes associated with location, data generated from one region may not be useful in another region. Also, improvements in grain legumes through breeding has led to development of cultivars low in ANFs (Cargo-Froom et al., 2023), presenting the possibility to increase use of grain legumes in broiler diets. For instance, zero-tannin faba bean cultivars have been developed (Woyengo and Nyachoti, 2012). Also, Hamungalu et al. (2026b) reported that levels of trypsin inhibitor activity and phytic acid in grain legumes were comparable to that of SBM.

The present study evaluated effects of inclusion at 100 or 200 g/kg of Australian-grown lupin and faba bean on growth performance, nutrient digestibility and gastrointestinal tract development of broilers fed starter diets.

## Materials and methods

The experiment was conducted according to the New Zealand Code of Ethical Conduct for the use of live animals for research, testing and teaching and approved by the Massey University (MUAC 22/82).

### Diets

Lupin and faba bean were sourced from a commercial supplier in Australia and ground through a 2.0 mm sieve. Five diets based on lupin or faba bean at 100 and 200 g/kg inclusion, or soybean meal (control diet) or were formulated to breeder’s recommendations (Table 1; Aviagen, 2022), using Allix^3^ feed formulation software (version 25.06; A-Systems, Versailles, France). The N-corrected apparent metabolizable energy **(AMEn)** and standardized ileal digestible amino acid values for test ingredients determined in our previous studies (Hamungalu et al., 2026a, 2026b) were used for diet formulation. Diets contained xylanase and phytase, and titanium dioxide at 5 g/kg (TiO2, Merck KGaA, Darmstadt, Germany) as an indigestible marker. The diets were steam-pelleted (conditioned at 75°C for 30 s) in a pellet mill (Model Orbit 15; Richard Sizer Ltd., Kingston-upon-Hull, UK) which has the capacity to manufacture 180 kg of feed/h and is fitted with a die ring, with specifications of 3.0 mm-holes and 35 mm-depth.

**Table 1:** Composition, calculated analysis and analyzed values (%, as fed basis) of broiler diets.

Item	SBM	10% Lupin	20% Lupin	10% Faba bean	20% Faba bean
Wheat 11%	62.28	58.87	52.68	58.49	49.76
Soybean meal 41.1%	31.03	21.13	15.10	21.87	18.71
Lupin		10.00	20.00		
Faba bean				10.00	20.00
Meat and bone meal	1.000	4.060	4.247	3.931	3.903
Soybean oil	1.329	2.444	4.396	2.133	4.139
Dicalcium phosphate	0.801				
Limestone	0.923	0.471	0.392	0.560	0.578
DL-Methionine	0.358	0.430	0.477	0.427	0.453
L-Lysine HCl	0.342	0.470	0.536	0.403	0.338
L-Threonine	0.195	0.253	0.279	0.253	0.252
L-Tryptophan		0.049	0.065	0.054	0.065
L-Valine	0.121	0.191	0.234	0.176	0.172
L-Isoleucine	0.112	0.186	0.224	0.181	0.184
L-Arginine	0.091	0.064	0.000	0.106	0.029
Sodium chloride	0.242	0.192	0.177	0.202	0.222
Sodium bicarbonate	0.247	0.256	0.272	0.247	0.217
Choline chloride	0.011	0.018	0.005	0.053	0.066
Xylanase^1^	0.005	0.005	0.005	0.005	0.005
Phytase^2^	0.010	0.010	0.010	0.010	0.010
Trace mineral premix^3^	0.100	0.100	0.100	0.100	0.100
Vitamin premix^3^	0.100	0.100	0.100	0.100	0.100
Pellet binder^4^	0.200	0.200	0.200	0.200	0.200
Titanium dioxide	0.500	0.500	0.500	0.500	0.500
**Calculated analysis**					
Crude protein	21.0	21.0	20.7	21.0	21.0
AMEn (Kcal/kg)	2900	2904	2906	2902	2900
Crude fat	2.91	4.53	6.73	3.90	5.82
Starch	38.02	35.72	31.89	38.97	37.17
Crude fiber	1.62	1.59	1.44	1.58	1.36
Total calcium	0.95	0.95	0.95	0.95	0.95
Total phosphorus	0.59	0.55	0.53	0.57	0.56
Non-phytate phosphorus	0.50	0.50	0.50	0.50	0.50
Sodium	0.18	0.18	0.18	0.18	0.18
Chloride	0.28	0.28	0.28	0.28	0.28
Potassium	1.04	0.87	0.77	0.92	0.91
Digestible lysine	1.27	1.27	1.27	1.27	1.27
Digestible methionine	0.67	0.69	0.71	0.70	0.71
Digestible methionine + cysteine	0.96	0.96	0.96	0.96	0.96
Digestible threonine	0.85	0.85	0.85	0.85	0.85
Digestible tryptophan	0.22	0.24	0.24	0.24	0.24
Digestible valine	0.95	0.95	0.95	0.95	0.95
Digestible isoleucine	0.84	0.84	0.84	0.84	0.84
Digestible leucine	1.37	1.26	1.19	1.27	1.27
Digestible arginine	1.34	1.34	1.34	1.34	1.34
**Analyzed values**^5^					
DM	89.8	89.8	90.7	90.4	90.8
Crude protein	22.7	23.0	22.4	23.0	22.6
Starch	33.7	31.4	28.1	34.0	33.4
Fat	4.43	6.79	5.76	5.17	8.07
Calcium	0.69	0.71	0.66	0.69	0.74
Phosphorus	0.59	0.60	0.56	0.59	0.57
Gross energy (Kcal/Kg)	3917	4013	4156	4013	4084

SBM, soybean meal.

1 ECONASE® XT 25 Xylanase 160 000 BXU/g, 38 - 150 g/tons of feed inclusion, AB Enzymes GmbH, Darmstadt, Germany.

2 Ronozyme HiPhos (1,000 phytase units [FYT]/kg diet). One FYT is defined as the activity of enzyme that releases 1.0 mmole of inorganic phosphorus/min from 5.0 mM sodium phytate at pH 5.5 at 37°C. Nutrient matrix values (0.15% non-Phytate P and 0.18% Ca) were used in basal diet formulation.

3 Supplied per kilogram of diet: antioxidant (ethoxyquin), 100 mg; biotin, 0.2 mg; calcium pantothenate, 12.8 mg; cholecalciferol, 0.06 mg; cyanocobalamin, 0.017 mg; folic acid, 5.2 mg; menadione, 4 mg; niacin, 35 mg; pyridoxine, 10 mg; trans-retinol, 3.33 mg; riboflavin, 12 mg; thiamine, 3.0 mg; dl-α-tocopheryl acetate, 60 mg; choline chloride, 638 mg; Co, 0.3 mg; Cu, 3.0 mg; Fe, 25 mg; I, 1 mg; Mn, 125 mg; Mo, 0.5 mg; Se, 0.2 mg; Zn, 60 mg.

4 KEMBIND® (Kemin Industries [Asia] Pte Ltd) pellet binder, which contained modified lignosulphonate, guar gum, edible fatty acids and mineral oil,was added on top of each diet.

5 Individual sample.

### Birds and housing

A total of 320, day-old male broilers (Ross 308) obtained from a commercial hatchery were individually weighed and randomly allocated to 40 pens (length 1.6 m x width 0.8 m) so that the average bird weight per pen was similar. Each pen had 8 birds, and 8 replicated pens per dietary treatment. Birds were raised in floor (wood shavings litter) pens until d-14 when they were moved to cages (length 0.6 m x width 0.6 m) to facilitate excreta collection for determination of apparent metabolizable energy (**AME**). Birds were housed in an environmentally controlled room supplying 20 h of fluorescent illumination per day. On day 1, the temperature was at 31°C and was then gradually reduced to 23°C by day 21. Fresh and clean water was readily available, and feed was provided *ad libitum* throughout the experiment.

### Pellet quality

Pellet durability index **(PDI)** of diets was determined in a Holmen Pellet Tester (New Holmen NHP100 Portable Pellet Durability Tester, TekPro Ltd., Willow Park, North Walsham, Norfolk, UK) as described by Abdollahi et al. (2010). Briefly, pellet samples (8 replicates per diet; 100 g each) without fines were rapidly circulated in an air stream around a perforated test chamber for 30 s. During the test, fines were removed continuously through a 2.0 mm sieve. Pellets were ejected and weighed manually at the end of test cycle. The PDI was calculated as the ratio of pellets not passing through the 2.00 mm sieve after test to whole pellets at the start.

Pellet hardness of diets was determined in a Kahl Pellet Tester (Hercules L, AMANDUS KAHL GmbH andCo. KG, Dieselstraße, Reinbek, Germany) using the method described by Abdollahi et al. (2010). Fifteen pellets of similar size were selected from each diet and individually inserted between a pressure piston and bar. Force (newton) needed to break pellets was determined by increasing the pressure applied by means of the pressure piston.

### Performance data

Mortality was recorded daily during the experimental period. Feed intake and body weight of birds were recorded on a pen/cage basis at weekly intervals. The FCR was corrected for body weight of any dead bird.

### Determination of metabolizable energy

Feed intake and excreta output for each cage were recorded over 4 consecutive days from d 18 to 21 post-hatch. Excreta were pooled daily by cage, frozen, and mixed in a blender. Representative samples were obtained and freeze-dried (Model 0610, Cuddon Engineering, Blenheim, New Zealand). Excreta samples were ground to pass through a 0.5 mm sieve and stored in airtight plastic containers at 4°C pending analysis of dry matter (**DM**), nitrogen (**N**) and gross energy (**GE**).

### Determination of the coefficient of apparent ileal digestibility

On d-21, birds from each cage with body weights closest to the mean of the cage were euthanised by intravenous injection (1 mL per 2 kg live weight) of sodium pentobarbitone (Provet NZ Pty Ltd., Auckland, New Zealand), and ileal digesta was collected from the lower half of the ileum as described by Ravindran et al. (2005). The ileum was defined as that portion of the small intestine extending from Meckel’s diverticulum to a point 40 mm proximal to the ileo-caecal junction. Digesta from the lower ileum of each bird was gently flushed into a plastic container with distilled water, pooled by cage, frozen, and freeze dried. Diets and freeze dried digesta were ground to pass through a 0.5 mm sieve and stored in airtight containers at 4°C pending laboratory analysis. Ileal digesta samples and diets were analyzed for DM, N, GE, calcium **(Ca)**, phosphorus **(P)**, fat, starch and titanium **(Ti)**.

### Digestive tract measurements and gizzard pH

Two birds euthanised for ileal collection from each cage, were used for measurements of the gastrointestinal tract. The proventriculus, gizzard, small intestine and ceca were excised, had adherent fat and mesentery removed, and were emptied of digesta. The empty digestive tract sections were rinsed, dried, and weighed. Before being emptied, pH of gizzard contents was measured as described by Singh et al. (2014). For each bird, gizzard pH was measured at 3 different regions (proximal, middle and distal) by inserting a calibrated digital pH meter (pH spear, Oakton Instruments, Vernon Hill, IL).

### Intestine morphology

Intestine morphology was measured as described by Naderinejad et al. (2016). From the two birds from each replicate euthanised for ileal collection, a middle section (about 5 cm in length) of jejunum and ileum were excised and flushed with cold saline and immediately placed in 10% formalin solution. Samples were transferred to 70% ethanol after 72 h. Each fixed sample was then processed on a tissue processor. Samples were dehydrated through graded alcohol concentrations (70%, 95% and absolute alcohol) at ambient temperature, cleared in graded concentrations of isopropyl alcohol to remove any residual alcohol, and then impregnated with Histosec pastilles under pressure at 60°C. Samples were embedded in wax and cut using a rotary microtome to a thickness of 5 μm using Feather S35 disposable blades. Samples were then stained with alcian blue and hematoxylin-eosin and examined by light microscopy. Two segments were fixed in each slide per bird, and slides were viewed (samples were coded) on an Olympus microscope (BX51TF, Olympus, Tokyo, Japan). Villus height (distance from apex of the villus to the junction of villus and crypt) and crypt depth (distance from the junction to the basement membrane of the epithelial cell at the bottom of the crypt) were measured. Visual measurements of villus height and crypt depth were made on 10 villi at 4 × magnification using microscopy imaging software (cellSens Standard [Ver.1.18] Olympus, Tokyo, Japan).

### Moisture content of excreta

Moisture content of excreta was determined using the oven dry method to assess litter quality. Excreta output collected for each cage was recorded over 4 consecutive days from d 18 to 21 post-hatch. Excreta were pooled by cage daily, frozen, mixed in a blender, and representative 20 g samples for each replicate was obtained and placed in an oven to dry at 110°C for 24 hours (Kheravii et al., 2017).

### Cost analysis

Analysis of feed cost was based on ingredient costs (Australian dollar) obtained at the time of diet preparation. Other costs were assumed similar across groups, since feed processing and bird management were not different.

### Chemical analysis

The DM of diets, ileal digesta and excreta were determined using standard procedures (Methods 930.15 and 925.10; AOAC, 2016). The GE was determined by adiabatic bomb calorimetry (Gallenkamp Autobomb, Weiss Gallenkamp Ltd, Loughborough, UK) standardized with benzoic acid. Starch content was determined using a Megazyme kit (Method 996.11; AOAC, 2016) based on thermostable α-amylase and amyloglucosidase. Nitrogen was determined by combustion (Method 968.06; AOAC, 2016) using a carbon nanosphere-200 carbon, N and sulphur autoanalyzer (LECO Corporation, St. Joseph, MI). Crude fat was determined by the Soxhlet extraction method (Method 991.36; AOAC, 2005). Ca and P were determined by colorimetric methods following ashing of samples at 550°C and acid digestion in 6.0 M HCl (Method 968.08D; AOAC, 2005). Diet and ileal digesta samples were tested for Ti on a UV spectrometer in accordance with the method described by Short et al. (1996).

## Calculations

All data were expressed on a DM basis, and AME was determined using the following formulae (Khalil et al., 2023):

$$\text{AM}E_{\text{Diet}}\left(\text{Kcal}/\text{kg}\right)\;=\;\left[\left(\text{FI}\;\times \;GE_{\text{Diet}}\right)\;-\;\left(\text{Excreta}\;\text{output}\;\times GE_{\text{Excreta}}\right)\right]\;/\;\text{FI}$$

Where, FI = feed intake, GE_diet_ = GE of the diet, and GE_excreta_ = GE of the excreta.

Nitrogen retention, as a percentage of intake, was determined as follows:

$$N_{\text{retention}\;\left(\%\right)}\;=\;100\;\times \;\left[\left(\left(\text{FI}\;\times \;N_{\text{diet}\;}\right)\;-\;\left(\text{Excreta}\;\text{output}\;\times \;N_{\text{excreta}}\right)\right)\;/\;\left(\text{FI}\;\times \;N_{\text{diet}}\right)\right]$$

Where, N_diet_ = N of the diet, and N_excreta_ = N of the excreta.

Determination of AMEn was by correcting for zero N retention by multiplication with 8.73 Kcal per g N retained in the body as described by Titus et al. (1959).

Coefficient of apparent ileal digestibility (**CAID**) of nutrients was calculated using the following formulae (Hamungalu et al., 2020):

$$\text{CAID}\;\text{of}\;\text{nutrient}=\left[{\left(\text{Nutrient}/\text{Ti}\right)}_{d}-\;{\left(\text{nutrient}/\text{Ti}\right)}_{i}\right]/{\left(\text{nutrient}/\text{Ti}\right)}_{d}$$

$$\text{Where},\;{\left(\text{nutrient}\;/\;\text{Ti}\right)}_{d}=\text{ratio}\;\text{of}\;\text{nutrient}\;\text{to}\;\text{Ti}\;\text{in}\;\text{the}\;\text{diet},\;\text{and}\;{\left(\text{nutrient}\;/\;\text{Ti}\right)}_{i}\;=\text{ratio}\;\text{of}\;\text{nutrient}\;\text{to}\;\text{Ti}\;\text{in}\;\text{the}\;\text{ileal}\;\text{digesta}.$$

The moisture content of excreta was calculated as follows (Barker et al., 2013):

$$\text{MC}=\left(\frac{M_{w}-M_{d}}{M_{w}-M_{t}}\right)\times \;100$$

Where, MC = moisture content (%), Mw = mass of the wet solids (including the oven dish) (g), Md = mass of the dry solids (including the oven dish) (g) and Mt = mass of the oven dish (g)

Cost of feed per bird and weight gain were calculated as follows (Kandel et al., 2025);

$$\text{Feed}\;\text{cost}\;\text{per}\;\text{kg}\;\text{of}\;\text{weight}\;\text{gain}=\frac{\text{Cost}\;\text{per}\;\text{bird}\;\text{at}\;21\;d}{\text{Weight}\;\text{gain}\;\text{per}\;\text{bird}}\times \;1000$$

$$\text{Cost}\;\text{per}\;\text{bird}\;\text{at}\;21\;d=\frac{\text{Cost}\;\text{of}\;\text{feed}\;x\;\text{Feed}\;\text{intake}\;\text{for}\;21\;d\;\text{period}\;}{1000}$$

### Statistical analysis

Data was analyzed using the General Linear Models procedure of SAS (version 9.4; 2015. SAS Institute Inc., Cary, NC) using one-way ANOVA. Pen or cage served as experimental unit. Differences were considered significant at *P* *<* 0.05 and significant differences were separated by Tukey’s test.

## Results

### Pellet quality

Pellet hardness and PDI of the SBM-based diet were greater (Table 2) than that of diets with grain legumes. The diet containing 200 g/kg of grain legume had the lowest pellet hardness and PDI.

**Table 2:** Effect of feeding diets containing lupin and faba bean on weight gain (WG; g/bird), feed intake (FI; g/bird), feed conversion ratio (FCR; g feed/g gain), and feed cost (AUD; Australian dollars) for broilers,^1^ and pellet quality of feed.

Days post-hatch	Item	Diet					SEM	*P-*value
		SBM	10% Lupin	20% Lupin	10% Faba	20% Faba		
1 – 7	WG	168^b^	182^a^	182^a^	177^ab^	179^ab^	3.02	0.017
	FI	166	174	167	168	167	2.89	0.321
	FCR	0.988^a^	0.953^b^	0.918^c^	0.950^b^	0.936^bc^	0.007	0.001
8 – 14	WG	454^bc^	470^ab^	472^a^	451^c^	449^c^	4.46	0.001
	FI	525^ab^	532^a^	514^abc^	504^bc^	496^c^	6.13	0.001
	FCR	1.158^a^	1.131^b^	1.105^c^	1.119^bc^	1.103^c^	0.004	0.001
15 – 21	WG	611^b^	652^a^	625^b^	604^b^	610^b^	6.09	0.001
	FI	807^a^	805^ab^	778^bc^	766^cd^	740^d^	6.89	0.001
	FRC	1.320^a^	1.236^b^	1.232^b^	1.247^b^	1.215^b^	0.009	0.001
1 – 21	WG	1243^b^	1301^a^	1286^a^	1232^b^	1240^b^	9.48	0.001
	FI	1483^ab^	1508^a^	1460^b^	1439^bc^	1409^c^	11.0	0.001
	FCR	1.213^a^	1.161^b^	1.139^cd^	1.157^bc^	1.135^d^	0.005	0.001
								
Pellet durability index (%)^2^		83.9^a^	80.4^b^	60.1^d^	76.3^c^	58.9^d^	0.49	0.001
Pellet hardness (Newton)^3^		9.61^a^	9.42^a^	8.37^b^	9.22^a^	8.31^b^	0.15	0.001
							
Feed cost per bird and kg of weight gain								
1 – 21	AUD/bird	1.45^c^	1.57^b^	1.65^a^	1.46^c^	1.50^c^	0.014	0.001
	AUD/kg WG	1.17^c^	1.21^bc^	1.29^a^	1.18^c^	1.22^b^	0.009	0.001

1 Data are mean values of 8 replicate pens per treatment (8 birds per replicate pen). SBM, soybean meal; SEM, standard error of the mean.

2 Each value represents the mean of 8 replicate samples.

3 Each value represents the mean of 15 replicate samples. ^a-d^Means in a row not sharing a common superscript are different (*P<* 0.05).

### Growth performance

A mortality rate of 1.56% was recorded during the whole trial period and was not related to dietary treatments. During the period from d-1 to 7, birds fed diets containing 100 or 200 g/kg lupin had greater WG and lower FCR than those fed the SBM based diet (*P* *<* 0.05; Table 2). The FCR of birds fed faba bean diets were lower (*P* *<* 0.05) than that of SBM, but WG and FI were not different (*P* *>* 0.05).

During the period from d 8 to 14, WG of birds fed the 200 g/kg lupin diet was greater (*P* *<* 0.05; Table 2) than that of birds fed faba bean or SBM diets. Birds fed SBM had more efficient FCR (*P* *<* 0.05) than those fed grain legumes. For the period 15 - 21 d, birds fed the 100 g/kg lupin diet had greater (*P* *<* 0.05) WG than birds fed SBM or faba bean diets. Birds fed the SBM diet had the most efficient FCR (*P* *<* 0.05).

Over the entire trial period (d-1 to 21), birds fed diets containing lupin had greater WG (*P* *<* 0.05; Table 2) than those fed faba bean or SBM diets. Birds fed the 200 g/kg faba bean diet had lower FI (*P* *<* 0.05) than birds fed SBM or lupin diets. Feed cost per bird and kilogram of WG were greatest in lupin fed birds (Table 2).

### Nutrient digestibility and energy utilization

The CAID of DM was lowest (*P* *<* 0.05; Table 3) in SBM and 200 g/kg lupin diet. SBM diet had lower CAID of N (*P* *<* 0.05) compared to diets containing grain legumes, except that the diet containing 100 g/kg faba bean was not different (*P* *>* 0.05). The CAID of starch was greater (*P* *<* 0.05) in lupin diet than in SBM or faba bean diets. Fat digestibility in SBM diet was lower (*P* *<* 0.05) than that of 200 g/kg faba bean. The CAID of Ca in diets containing 200 g/kg lupin or faba bean was greater than that in the SBM diet (*P* *<* 0.05). The ileal digestible energy (**IDE**) was lowest (*P* *<* 0.05) in SBM diet. The AME and AMEn were greatest (*P* *<* 0.05) in the 200 g/kg faba bean diet, and lowest (*P* *<* 0.05) in the SBM diet, while faba bean diets had greater (*P* *<* 0.05) GE metabolizability than lupin or SBM diets.

**Table 3:** Effect of feeding lupin and faba bean containing diets on the coefficient of apparent ileal digestibility (CAID) of dry matter (DM), nitrogen (N), starch, fat, calcium (Ca), and phosphorous (P), and ileal digestible energy (IDE; Kcal/Kg, DM), apparent metabolizable energy (AME; Kcal/kg, DM), N-corrected AME (AMEn; Kcal/kg, DM), and gross energy (GE) in broilers_._

	CAID^1^						Energy utilization^2^			
Diet	DM	N	Starch	Fat	Ca	P	IDE^1^	AME	AMEn	GE^3^
SBM	0.708^b^	0.830^c^	0.969^b^	0.928^b^	0.397^b^	0.657	3272^b^	3234^c^	3004^c^	0.688^b^
10% Lupin	0.733^ab^	0.865^ab^	0.990^a^	0.936^ab^	0.521^ab^	0.686	3436^a^	3344^b^	3093^b^	0.693^b^
20% Lupin	0.710^b^	0.880^a^	0.995^a^	0.923^b^	0.562^a^	0.707	3466^a^	3394^b^	3149^b^	0.686^b^
10% Faba	0.746^a^	0.853^bc^	0.970^b^	0.923^b^	0.429^ab^	0.680	3457^a^	3403^b^	3152^b^	0.709^a^
20% Faba	0.760^a^	0.875^ab^	0.973^b^	0.953^a^	0.583^a^	0.723	3565^a^	3484^a^	3238^a^	0.722^a^
SEM	0.008	0.006	0.002	0.006	0.039	0.020	32.3	16.5	15.1	0.003
*P-*value	0.001	0.001	0.001	0.005	0.005	0.209	0.001	0.001	0.001	0.001

^a-c^Means in a column not sharing a common superscript are different (*P<* 0.05). SBM, soybean meal; SEM, standard error of the mean.

1 Data are mean values of 8 replicate cages per treatment (8 birds per replicate cage), measured on day-21 (CAID).

2 Data are mean values of 8 replicate cages per treatment (8 birds per replicate cage), measured from 18 to 21 days post-hatch.

3 GE metabolizability = AMEn / GE content of the diet.

Coefficient of apparent ileal indigestibility **(CAII)** of N was lowest (*P* *<* 0.05; Table 4) in 200 g/kg lupin and greatest in SBM diet. Lupin had a lower (*P* *<* 0.05) CAII of starch than other treatments. The CAII of fat in SBM was not different (*P* *>* 0.05) from that of grain legume diets. The CAII of Ca was greatest in SBM, and lowest in 200 g/kg of lupin and faba bean. CAII of P was not different (*P* *>* 0.05) among treatments.

**Table 4:** Effect of feeding lupin and faba bean containing diets on the coefficient of ileal indigestibility and indigestible content (g/kg feed, as received basis) of Nitrogen (N), crude protein (CP), starch, fat, calcium (Ca) and phosphorus (P)^1^.

	Indigestibility coefficient							Indigestible content		
Diet	N	Starch	Fat	Ca	P	CP	Starch	Fat	Ca	P
SBM	0.170^a^	0.031^a^	0.072^a^	0.603^a^	0.343	38.5^a^	10.5^a^	3.20	4.19^a^	2.03
10% Lupin	0.135^bc^	0.010^b^	0.064^ab^	0.480^ab^	0.314	31.1^bc^	3.06^b^	4.37	3.41^ab^	1.89
20% Lupin	0.120^c^	0.005^b^	0.077^a^	0.439^b^	0.293	26.9^c^	1.41^b^	4.43	2.89^b^	1.65
10% Faba	0.147^ab^	0.030^a^	0.077^a^	0.571^ab^	0.321	33.9^ab^	10.1^a^	3.99	3.94^ab^	1.89
20% Faba	0.125^bc^	0.027^a^	0.047^b^	0.417^b^	0.277	28.2^c^	9.12^a^	3.77	3.08^b^	1.58
SEM	0.006	0.002	0.006	0.039	0.020	1.32	0.82	0.37	0.27	0.12
*P-*value	0.001	0.001	0.005	0.005	0.210	0.001	0.001	0.147	0.007	0.067

^a-c^Means in a column not sharing a common superscript are different (*P<* 0.05). SBM, soybean meal; SEM, standard error of the mean.

1 Data are mean values of 8 replicate cages per treatment (8 birds per replicate cage), measured on day 21.

Indigestible content of CP and Ca were greatest (*P* *<* 0.05; Table 4) in SBM, and lowest in 200 g/kg lupin and faba bean. Lupin had a lower (*P* *<* 0.05) indigestible content of starch than other treatments. The indigestible content of fat and P were not different among treatments.

### Digestive tract measurement

Apart from the 100 g/kg faba bean diet, birds fed grain legumes had greater (*P* *<* 0.05) relative empty weight of gizzard and proventriculus, but there was no difference (*P* *>* 0.05) for small intestine and ceca (Table 5). Birds fed the diet containing 100 g/kg faba bean had shorter (*P* *<* 0.05) relative length of jejunum and ileum than that of birds fed the SBM diet. Relative length of ceca for birds fed the SBM diet was not different from those fed grain legumes, except that bird fed the 200 g/kg lupin diet had shorter (*P* *<* 0.05) ceca than those fed the SBM diet. Gizzard pH in birds fed the SBM diet was not different (*P* *>* 0.05) to than in birds fed grain legumes, except that pH was more acidic in birds fed the 200 g/kg faba bean diet (Table 5).

**Table 5:** Influence of lupin and faba bean on excreta moisture content, gizzard pH, relative empty weight (g/kg body weight) and length (cm/kg body weight) of digestive tract segments in 21-day old broilers.

	Relative weight						Relative length				Giz. pH	Moisture
	Giz	Prov	Duo	Jej	Ile	Cec	Duo	Jej	Ile	Cec		
SBM	8.9^c^	2.7^c^	5.1	9.4	6.8	2.5	24.1	60.4^a^	67.5^a^	13.8^a^	3.22^a^	76.5^a^
10% Lupin	10.4^ab^	3.1^a^	5.7	10.1	6.6	2.4	23.8	58.6^ab^	61.7^ab^	13.0^ab^	2.90^ab^	75.1^ab^
20% Lupin	10.8^ab^	3.0^ab^	5.6	9.5	7.1	2.5	23.0	56.0^ab^	62.1^ab^	12.6^b^	2.74^ab^	75.0^ab^
10% Faba	9.6^bc^	2.7^bc^	5.0	8.8	6.1	2.4	24.2	53.2^b^	59.8^b^	13.0^ab^	2.92^ab^	74.5^b^
20% Faba	11.1^a^	3.2^a^	5.1	9.1	6.0	2.7	24.1	56.2^ab^	60.0^b^	13.9^a^	2.61^b^	73.7^b^
SEM	0.34	0.08	0.22	0.39	0.33	0.08	0.48	1.46	1.67	0.26	0.136	0.47
*P-*value	0.001	0.001	0.141	0.206	0.115	0.140	0.383	0.016	0.017	0.005	0.038	0.004

^a-c^Means in a column not sharing a common superscript are different (*P≤* 0.05).

SBM, soybean meal; SEM, standard error of the mean; Giz, gizzard; prov, proventriculus; duo, duodenum; jej, jejunum; cec, caeca.

### Small intestinal morphology and moisture content of excreta

Moisture content of excreta was highest (*P* *<* 0.05; Table 5) and lowest (*P* *<* 0.05) for birds fed diets containing SBM and faba bean, respectively. There were no differences (*P* *>* 0.05; Table 6) among treatments regarding villi height and crypt depth.

**Table 6:** Effect of feeding diets containing lupin and faba bean on villi height (µm), crypt depth (µm) and, villus to crypt depth ratio of jejunum and ileum in 21-day old broilers.

Diet	Jejunum			Ileum		
	Villus height	Crypt depth	Villus/crypt	Villus height	Crypt depth	Villus/crypt
Soybean meal	1278	173	7.43	714	120	6.00
10% Lupin	1359	164	8.30	644	127	5.19
20% Lupin	1202	153	8.04	719	132	5.57
10% Faba bean	1252	160	7.97	638	130	4.95
20% Faba bean	1277	172	7.60	706	136	5.21
SEM	46.4	7.33	0.44	27.9	6.36	0.30
*P*-value	0.224	0.269	0.636	0.110	0.533	0.130

SEM, standard error of the mean.

## Discussion

Pellet quality of a diet influences growth performance of broilers (McKinney and Teeter, 2004). Pellet hardness and PDI are the primary measures of pellet quality (Abdollahi et al., 2010; Muramatsu et al., 2015). In the present study, PDI and pellet hardness of the 200 g/kg grain legume diets were low, possibly due to high oil content. Lupin and faba bean were low in energy (Hamungalu et al., 2026a), therefore, soybean oil was added to their diets to balance for energy. Kandel et al. (2025) reported low PDI for diets high in oil. When Briggs et al. (1999) varied dietary oil content from 29 to 75 g/kg, PDI reduced from 91.2 to 62.9%. Also, the effect of oil on pellet quality depends on application site, either mixer-added or post-pellet added (Muramatsu et al., 2015). In Pope et al. (2018), PDI was lower when fat was mixer-added (90.5%) than when added post-pelleting (95.3%) for broiler starter diets. In the present study, soybean oil was added in the mixer (pre-conditioning), however, pellet quality may improve in diets containing a large inclusion of grain legumes if oil is applied post-pelleting. Post-pellet application of oil uses more electrical energy due to increased friction in the pellet die (Gehring et al., 2011; Pope et al., 2018). Poor pellet quality results when oil partially covers feed particles, thus, limiting steam penetration and lessening diet compaction through the pellet die (Muramatsu et al., 2015).

Over the whole trial period, growth performance of birds fed grain legumes was similar or greater than that of birds fed the SBM diet. This agreed with published findings with lupin (Nalle et al., 2011, 2012; Ravindran et al., 2002) and faba bean (Ivarsson and Wall, 2017; Nalle et al., 2010b) in pelleted broiler diets. On the other hand, Kaczmarek et al. (2016) and Viveros et al. (2007) reported reduced WG and poor FCR for birds fed mash diets containing 200 g/kg lupin. Also, Breytenbach and Ciacciariello (2006) reported poor growth rate of birds fed a diet containing 170 g/kg lupin for 21-d post-hatch. Kopmels et al. (2020) observed a reduction in average daily gain for the 12-d old broilers fed a mash diet containing 150 g/kg of faba bean. Similarly, Koivunen et al. (2015) observed a linear decrease in WG and FI with increasing faba bean inclusion (0, 80, 160, 240 g/kg) in broiler grower diets (cold-pelleted). Two factors could partly account for discrepancies in study outcomes, firstly is feed form. Gous (2011) reported that inclusion of faba bean in mash diets reduced WG and feed efficiency, but not in pelleted diets. Similar observations were made by Ivarsson and Wall (2017) who attributed the advantage of a pelleted diet to increased FI. They reported FI reduction of 26.4 and 10.0% in mash and pelleted starter diets containing 200 g/kg faba bean, respectively. Farrell et al. (1999) reported lower FI and WG for birds fed a cold pelleted (45°C) diet compared to those fed a steam-pelleted (70 – 80°C) diet. Secondly, differences in the approach to diet formulation can influence results, particularly when balancing for energy and AA in diets (Nalle et al., 2012). In Smit et al. (2021), a reduction in average daily gain by 40-d old broilers fed a faba bean-based diet was attributed to over-estimation of AA digestibility during diet formulation. Nutritional values of lupin and faba bean generally vary with cultivar (Kaczmarek et al., 2014; Nalle et al., 2011, 2012), as such, reliable nutritional information of grain legumes is important to better understand their effect on bird performance.

The FI of birds fed a diet containing 200 g/kg faba bean was lower than those fed the SBM diet, agreeing with findings of Koivunen et al. (2015). Koivunen et al. (2015) observed a linear decrease of FI and WG with increasing faba bean inclusion (80, 160 and 240 g/kg). Some faba bean cultivars contain tannin (Koivunen et al., 2015; Longstaff and McNab, 1991; Sadr et al., 2026) that has poor palatability which reduces FI (Ortiz et al., 1994). The effect of tannin is dose-dependent (Ortiz et al., 1994), therefore, a high level of tannin-containing faba bean in diets might have a deleterious effect on bird performance. In Kopmels et al. (2020), inclusion of tannin-free faba bean at 150, 300, and 400 g/kg in a starter, grower, and finisher diet, respectively, had no effect on FI. Australian grown grain legumes were used in the present study, but their cultivar information was not available, and tannin was not measured. A recent study using Australian grown faba bean reported 10 g/kg condensed tannin in the cultivar used (Sadr et al., 2026).

The greater WG and improved FCR in birds fed diets with lupin when compared to SBM-fed birds might be due to better nutrient utilization with lupin. Hamungalu et al. (2026b) and Kozłowski et al. (2011) reported that AA and N digestibility in lupin were similar or greater than with SBM. Also, in the present study, starch and N digestibility was greater in birds fed lupin diets, probably due to a well-developed gizzard and proventriculus. A developed gizzard is generally associated with improved nutrient utilization (Amerah et al., 2009; Hetland et al., 2003). Insoluble fiber stimulates gizzard development (Abdollahi et al., 2019; Amerah et al., 2009; Gonzalez-Alvarado et al., 2007; Hetland et al., 2003; Jimenez-Moreno et al., 2009), and lupin is relatively high in dietary fiber (Hamungalu et al., 2026a; Nalle et al., 2011). In the present study, diets containing 200 g/kg grain legumes had greater AMEn than those fed 100 g/kg grain legume. This agreed with Koivunen et al. (2015), who observed a linear increase in AMEn with increasing inclusion of faba bean. They attributed this to a greater amount of rapeseed oil added to diets. In the present study, more soybean oil was added to 200 g/kg grain legumes diets than to the 100 g/kg grain legume diets to balance for energy.

Villi height and crypt depth was not influenced by inclusion of grain legumes in starter diets, in agreement to Konieczka and Smulikowska (2018). In addition, moisture content of excreta from birds fed grain legumes was similar or lower than that of birds fed SBM. This agreed to findings of Ivarsson and Wall (2017), who observed no difference in DM of excreta between SBM and faba bean diets (100, 200, and 300 g/kg inclusion). Nalle et al. (2010a, 2012) observed no differences in excreta score between the 200 g/kg lupin cultivars and SBM diets. However, Breytenbach and Ciacciariello (2006) reported wet sticky droppings for birds fed 170 g/kg lupin, which they associated to soluble non-starch polysaccharides (**NSP**). In Farrell et al. (1999), viscosity and excreta stickiness increased with increasing lupin (120 - 360 g/kg) in starter broiler diets. Annison et al. (1996) reported that viscosity of digesta increased from 3.3 to 9.5 mPas·s when lupin (containing 218 and 27 g/kg insoluble and soluble NSP, respectively) was added to broiler diets. In Hejdysz et al. (2018), a negative association was observed between excreta DM and soluble NSP content in diets fed to broilers. The variable influence of lupin on excreta moisture occurs because the NSP content of lupin varies with species and cultivar (Hejdysz et al., 2018; Nalle et al., 2012). Smulikowska et al. (2014) reported more viscous digesta from feeding sweet narrow-leafed lupin (*L. angustifolius*) than from sweet yellow lupin (*L. luteus*), 3.12 and 2.60 mPas·s, respectively. Similarly, Hejdysz et al. (2018) noted that increased inclusion of narrow-leafed lupin (50 – 250 g/kg) increased excreta viscosity, while no differences were reported when yellow or white lupin (*L. albus*) were fed.

Feed cost per kilogram of weight gain for birds fed SBM diet was not different from those fed 100 g/kg lupin and faba bean in the present study. Proskina and Cerina (2017) reported a reduction in feed cost per kilogram of live body weight by 15.2 – 36.7% due to inclusion of field pea and faba bean at 100 or 200 g/kg in broiler diet. The variation in feed cost between studies might be due to diet composition and local price of grain legumes.

## Conclusion

This study showed that inclusion of lupin or faba bean up to 200 g/kg in starter diets did not compromise bird performance and nutrient digestibility, however, the cost per kilogram weight gain for the 200 g/kg lupin or faba bean diet were high. Also, greater inclusion of grain legumes requires a large amount of supplementary oil to balance for energy, which might affect pellet quality and bird performance. Future studies could examine effects of greater grain legume inclusion and feeding through different phases until market weight.

## Author contributions

All authors (O. Hamungalu, M.R. Abdollahi, P.C.H. Morel, S. Liu, and T.J. Wester) meet the requirements for ethical authorship of scientific articles as specified by the publishers. O. Hamungalu was responsible for conceptualization, formal analysis, investigation, writing of the original draft and subsequent revisions. M.R. Abdollahi was responsible for conceptualization, funding acquisition, formal analysis, and reviewing. S, Liu assisted primarily with conceptualization, discussion and interpretation. P.C.H. Morel assisted with formal analysis, discussion and reviewing. T.J. Wester was the chief supervisor and responsible for project administration, discussion, interpretation, and review and editing.

## Ethics declaration

This study was conducted in accordance with the following guidelines for animal welfare and/or reporting: New Zealand Code of Ethical Conduct for the use of live animals for research, testing and teaching. This study was approved by the Massey University Animal Ethics Committee. (Approval No. MUAC 22/82). All authors (O. Hamungalu, M.R. Abdollahi, P.C.H. Morel, S. Liu, and T.J. Wester) meet the requirements for ethical authorship of scientific articles listed below. This statement confirms that each author made a substantial intellectual contribution to the study and accepts full accountability for its content. The researchers meet all four of the following requirements: 1. Substantial Contributions: Substantive input into the conception or design of the work, OR the acquisition, analysis, or interpretation of data. 2. Drafting or Reviewing: Writing the manuscript draft OR reviewing it critically for crucial intellectual content. 3. Final Approval: Reviewing and explicitly approving the exact, final version of the manuscript before it goes to press. 4. Accountability: Agreeing to be personally and publicly responsible for all aspects of the work, ensuring that any questions regarding the accuracy or integrity of the research are thoroughly investigated and resolved.

## Disclosures

The authors declare that they have no known competing financial interests or personal relationships that could have appeared to influence the work reported in this paper.
